# Indocyanine Green (ICG) Lymphography Is Superior to Lymphoscintigraphy for Diagnostic Imaging of Early Lymphedema of the Upper Limbs

**DOI:** 10.1371/journal.pone.0038182

**Published:** 2012-06-04

**Authors:** Makoto Mihara, Hisako Hara, Jun Araki, Kazuki Kikuchi, Mitsunaga Narushima, Takumi Yamamoto, Takuya Iida, Hidehiko Yoshimatsu, Noriyuki Murai, Kito Mitsui, Taro Okitsu, Isao Koshima

**Affiliations:** 1 Department of Plastic Surgery and Reconstructive Surgery, The University of Tokyo, Tokyo, Japan; 2 Department of Vascular Surgery, Saiseikai Kawaguchi Hospital, Saitama, Japan; 3 Department of Rehabilitation Medicine, Keio University, Tokyo, Japan; University of Texas, M.D. Anderson Cancer Center, United States of America

## Abstract

**Background:**

Secondary lymphedema causes swelling in limbs due to lymph retention following lymph node dissection in cancer therapy. Initiation of treatment soon after appearance of edema is very important, but there is no method for early diagnosis of lymphedema. In this study, we compared the utility of four diagnostic imaging methods: magnetic resonance imaging (MRI), computed tomography (CT), lymphoscintigraphy, and Indocyanine Green (ICG) lymphography.

**Patients and Methods:**

Between April 2010 and November 2011, we examined 21 female patients (42 arms) with unilateral mild upper limb lymphedema using the four methods. The mean age of the patients was 60.4 years old (35–81 years old). Biopsies of skin and collecting lymphatic vessels were performed in 7 patients who underwent lymphaticovenous anastomosis.

**Results:**

The specificity was 1 for all four methods. The sensitivity was 1 in ICG lymphography and MRI, 0.62 in lymphoscintigraphy, and 0.33 in CT. These results show that MRI and ICG lymphography are superior to lymphoscintigraphy or CT for diagnosis of lymphedema. In some cases, biopsy findings suggested abnormalities in skin and lymphatic vessels for which lymphoscintigraphy showed no abnormal findings. ICG lymphography showed a dermal backflow pattern in these cases.

**Conclusions:**

Our findings suggest the importance of dual diagnosis by examination of the lymphatic system using ICG lymphography and evaluation of edema in subcutaneous fat tissue using MRI.

## Introduction

Secondary lymphedema results in swelling of the limbs due to lymph retention following resection, radiotherapy, and lymph node dissection in cancer therapy. Lymphedema induces severe pathological conditions, including enlargement of the limbs, recurrent cellulitis, and lymphorrhea, and may markedly impair quality of life [1]–[5]. However, no curative treatment for lymphedema has been established because the pathology is not fully understood.

Early diagnosis and therapy after appearance of edema are very important for treatment of secondary lymphedema [6], [7]. However, simple methods [8] such as inspection and palpation are only useful for diagnosis of lymphedema after aggravation to stage 2 or higher. Diagnosis of stage 1 (initial and irregular edema) is very difficult using these approaches. Currently, lymphoscintigraphy is viewed as the main approach for diagnosis of lymphedema [9]–[14]. However, definite diagnosis of early-stage lymphedema is also difficult because edema progresses very slowly; appears not only immediately after cancer therapy, but also after several years (more than 10 years in some cases); and shows within-day variation in the early stage. Thus, treatment is often initiated after edema progresses to morbidity.

**Table 1:** Summary of cases in the study.

Case	Age	Gender	Upper limblymph- edema	Primary disease	Lymph node dissection	Radio- therapy	Lymph edema stage	Duration of lymph- edema (Month)	Conservative treatment
1	54	Female	Right	Breast cancer	+	+	1	6	-
2	49	Female	Right	Breast cancer	+	+	1	12	+
3	55	Female	Right	Breast cancer	+	+	1	25	+
4	35	Female	Right	Breast cancer	+	–	1	17	+
5	56	Female	Left	Breast cancer	+	+	1	8	+
6	66	Female	Right	Breast cancer	+	+	1	17	+
7	73	Female	Right	Breast cancer	+	–	1	10	+
8	57	Female	Left	Axillary liposarcoma	+	+	1	9	–
9	73	Female	Left	Breast cancer	+	–	1	6	+
10	75	Female	Left	Breast cancer	+	+	1	18	+
11	60	Female	Right	Breast cancer	+	+	1	10	+
12	81	Female	Left	Breast cancer	+	–	1	24	+
13	78	Female	Left	Breast cancer	+	–	1	14	+
14	62	Female	Left	Breast cancer	+	+	1	17	+
15	44	Female	Right	Breast cancer	+	+	1	10	+
16	50	Female	Left	Breast cancer	+	–	1	9	+
17	57	Female	Right	Breast cancer	+	+	1	10	+
18	61	Female	Right	Breast cancer	+	+	1	7	–
19	43	Female	Left	Breast cancer	+	–	1	12	+
20	81	Female	Left	Breast cancer	+	+	1	14	+
21	59	Female	Right	Breast cancer	+	+	1	7	+

Recent advances in imaging techniques have led to improved diagnosis of lymphedema. Magnetic resonance imaging (MRI) [15], computed tomography (CT) [16], [17], and lymphoscintigraphy^9–14^ are commonly used for this purpose, and the newer technique of fluorescence lymphography using indocyanine green (ICG) [18], [19] is increasingly being used. Yamamoto showed that findings on ICG lymphography changed from a linear to a splash pattern and then to a diffuse pattern as the severity of lymphedema aggravated [20]–[25]. However, the advantages and disadvantages of each of these methods for diagnosis of lymphedema have not been fully investigated and a correlation of the progression of lymphedema with imaging findings has not been established.

**Table 2:** Results of imaging diagnosis.

Case	Increase in circumference of the limb^a^	Pitting edema	MRI	CT	Lymphoscintigraphy	ICG
1	–	+	+	–	–	+
2	–	+	+	–	+	+
3	–	+	+	+	+	+
4	–	+	+	–	+	+
5	–	+	+	–	–	+
6	–	+	+	–	+	+
7	–	+	+	+	+	+
8	–	+	+	–	–	+
9	–	+	+	–	–	+
10	–	+	+	+	+	+
11	–	+	+	–	+	+
12	–	+	+	+	+	+
13	–	+	+	+	+	+
14	–	+	+	+	+	+
15	–	+	+	–	+	+
16	–	+	+	–	–	+
17	–	+	+	–	–	+
18	+	+	+	+	+	+
19	–	+	+	–	+	+
20	–	+	+	–	–	+
21	–	+	+	–	–	+

a 3-cm increase relative to the contralateral side.

**Table 3:** Sensitivity and specificity of each test.

	Sensitivity	Specificity
MRI	1	1
ICG lymphography	1	1
Lymphoscintigraphy	0.62	1
CT	0.33	1

In this study, we diagnosed secondary upper limb lymphedema at an early stage using these four imaging methods and we also performed histopathological examinations. The objectives of the study were to compare the diagnostic accuracy of the imaging methods and to investigate their usefulness for identification of early-stage lymphedema.

**Figure 1:**
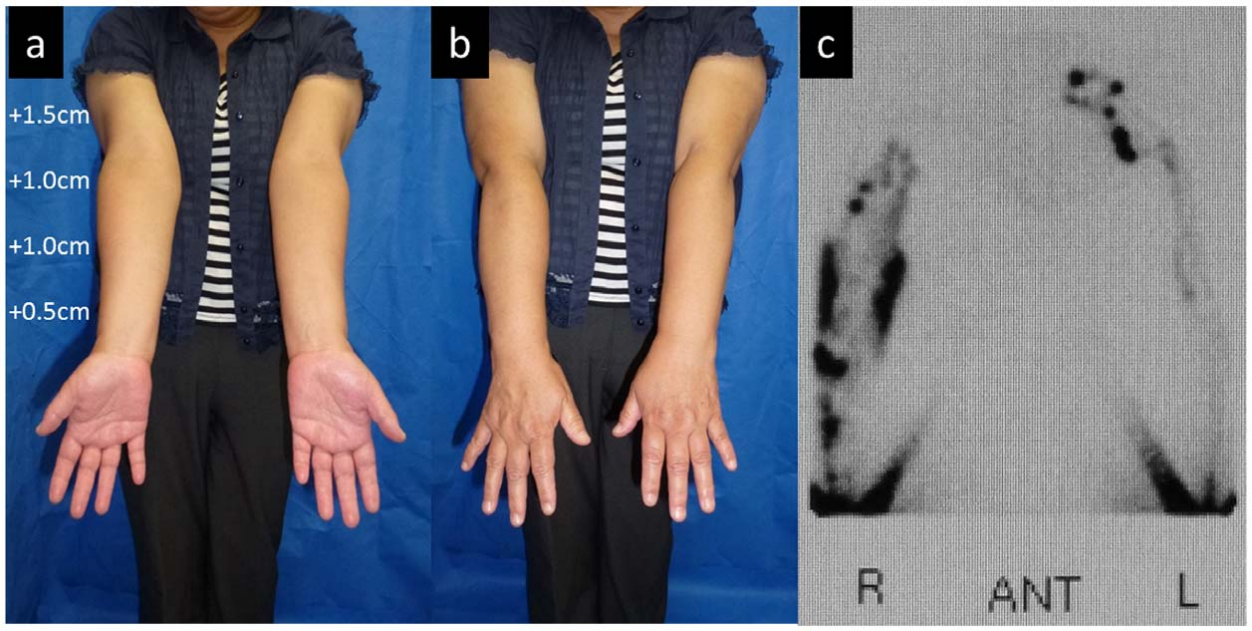
Case 3. (a, b) Edematous condition before LVA. Compared to the healthy side (left), the circumferences were increased by 1.5, 1.0, 1.0, and 0.5 cm for the upper arm, elbow, forearm, and wrist, respectively. (c) Findings from lymphoscintigraphy. Reflux in the skin (dermal backflow) was observed in the medial and lateral upper arm and lateral forearm in the right upper limb. A normal lymph vessel distribution was apparent in the left upper limb.

**Figure 2:**
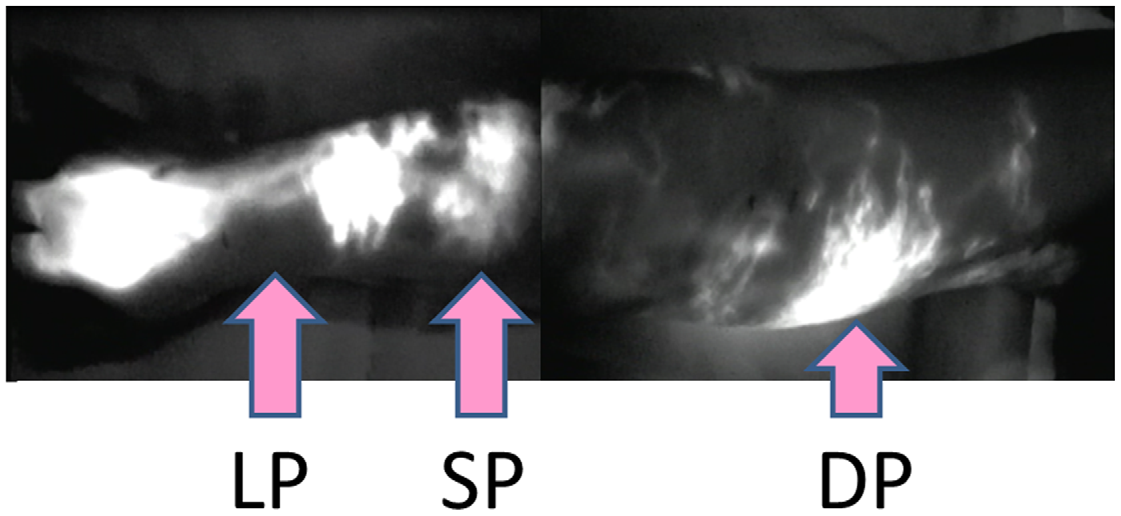
ICG lymphography findings in Case 3. Two linear patterns (LP) were noted in the wrist. A splash pattern (SP) was observed in the lateral forearm over the upper arm. Diffuse (DP) and splash patterns were mixed in some regions of the medial upper arm.

## Materials and Methods

### Patients

112 upper limb lymphedema patients (224 arms) visited our hospital between April 2010 and November 2011. We selected our patient as secondary lymphedema, ISL stage 1 without dropout. The subjects were 21 patients (42 arms) with unilateral mild upper limb lymphedema who were treated at our hospital (Table 1). Lymphedema staging was based on the International Society of Lymphology (ISL) classification and all patients were diagnosed as stage 1. In stage 1, accumulation of fluid that is relatively high in protein content occurs. There is visible swelling but this can be temporarily reduced by elevation of the limb. None of the patients had lymphedema in the contralateral arm. All the patients were female and their mean age was 60.4 years old (range: 35–81 years old). Resection of breast cancer with lymph node dissection had been performed in 21 patients, with postoperative radiotherapy in 14 of these cases. One patient had undergone resection of axillary liposarcoma with radiotherapy.

**Figure 3:**
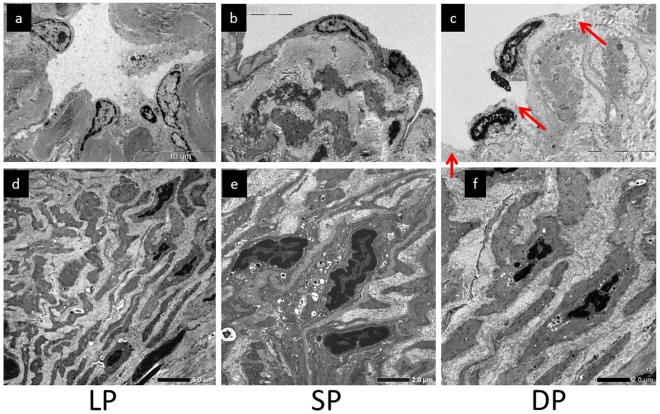
Case 3. Collecting lymph vascular endothelial cells in (a) linear pattern (LP), (b) splash pattern (SP), and (c) diffuse pattern (DP) areas. Regions in which endothelial cells were detached are indicated with red arrows. (d) ‘Contractile’ smooth muscle cells in (d) LP, (e) SP, and (f) DP areas. The space between smooth muscle cells was widened due to overgrowth of collagen fibers.

The mean duration of edema was 12.5 months (range: 6–25 months). All patients had received conservative treatment after the development of edema. The circumferences of the bilateral forearms (5 cm distal to the elbow) were measured in all 21 patients and an increase of ≥3 cm compared to the healthy side was regarded as positive. The bilateral upper limbs were examined using lymphoscintigraphy, ICG lymphography, MRI, and CT. The specificity and sensitivity were calculated by using the unaffected limbs as negative control. Lymphaticovenous anastomosis (LVA) was used to remit edema in 7 cases, in which collecting lymph vessels and skin were collected from the edematous region and evaluated histochemically using immunostaining and electron microscopy. The experiments performed in this study were conducted with the approval of the institution’s ethical committee, The university of Tokyo’s ethics committee and Saiseikai Kawaguchi general hospital’s ethics committee. And all the patients were provided written informed consent.

**Figure 4:**
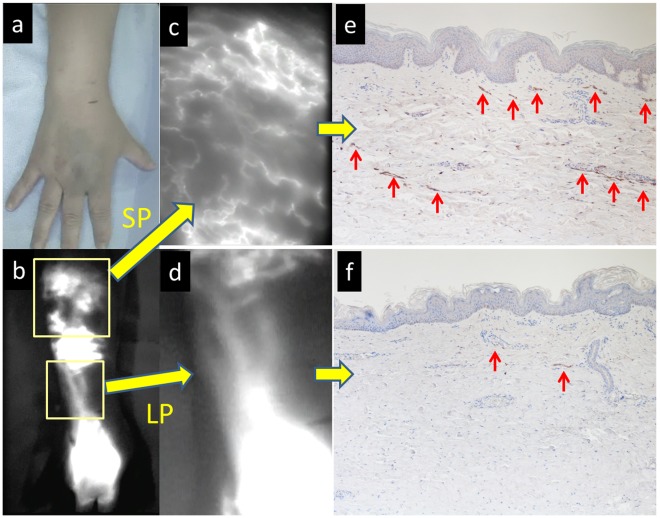
Case 3 . (a, b) Macroscopic and ICG lymphography findings in the dorsum of the hand over the forearm. (c) Magnified ICG lymphography findings in (c) splash pattern (SP) and (d) linear pattern (LP) areas. (e) Immunostaining of the skin in the SP area (LYVE-1). Overgrowth of subcutaneous capillary lymph vessels was apparent (red arrows). (f) Immunostaining of the skin in the LP area (LYVE-1). Skin capillary lymph vessels are diffusely present directly below the dermis (red arrows).

### Lymphoscintigraphy

A low-energy, high-resolution, collimator-equipped, dual-head, whole-body Millemium VG scinticamera (GE) was used for lymphoscintigraphy. With the patient in the supine position, a radioisotope, ^99m^Tc, was subcutaneously injected into the bilateral second interdigits (0.2 ml each, 0.4 ml in total, 80 MBq). Images of the whole affected arms were acquired using the scinticamera after 30, 60, 90, and 120 minutes. After subcutaneous injection, the injected regions were mildly massaged, but no exercise load was applied to the four limbs. Observation of dermal backflow of the radioisotope was taken as an indication of lymphedema.

**Figure 5:**
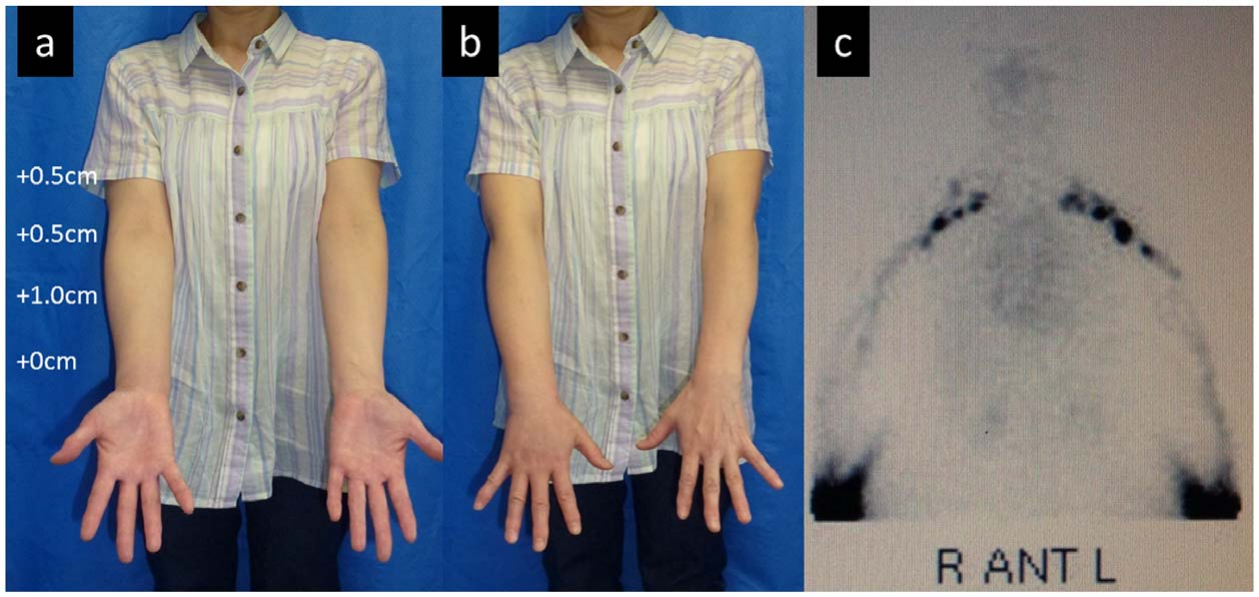
Case 17. (a, b) Clinical presentations. The circumferences were increased by 0.5, 0.5, 1.0, and 0 cm in the upper arm, elbow, forearm, and wrist, respectively, compared to the healthy side (left). (c) Findings from lymphoscintigraphy. A normal lymph vessel distribution was found in both upper limbs.

**Figure 6:**
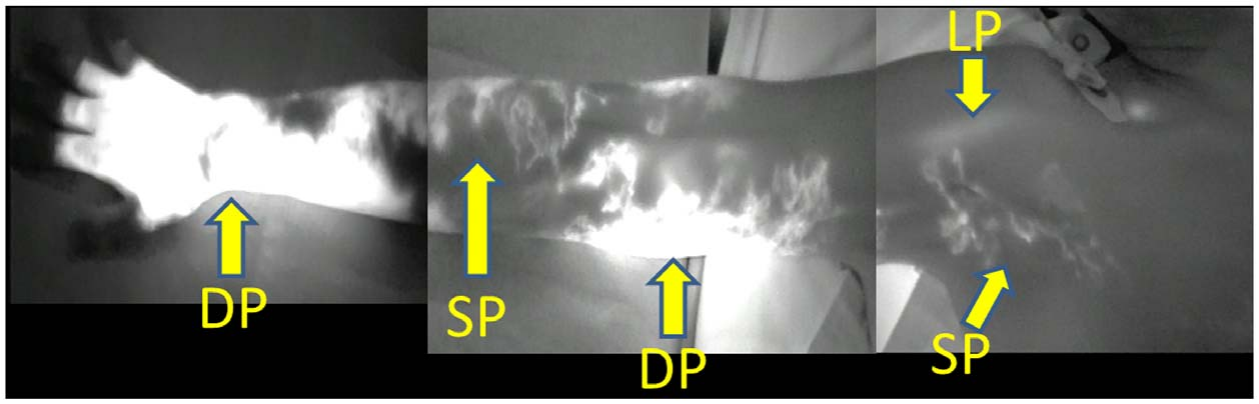
ICG lymphography findings in Case 17. A linear pattern (LP) was noted in the shoulder. A splash pattern (SP) was present in the lateral forearm over the upper arm, axilla, and precordia. In the medial upper arm, diffuse (DP) and splash patterns were mixed in some regions.

### ICG Lymphography

ICG (0.2 ml; Diagnogreen 0.5%, Daiichi Pharmaceutical, Tokyo, Japan) was injected intracutaneously into the bilateral second interdigits. Imaging was performed using a PDE system (Hamamatsu Photonics, Hamamatsu, Japan). Vivid dynamic images of superficial lymphatic flow were obtained within a few minutes after the injection. In this method, the skin surface is illuminated with near infrared light, and light emitted by ICG absorbed in lymph vessels is imaged using a CCD camera to detect lymph flow in subcutaneous lymph vessels. Although there are individual variations, linear lymph vessels in normal arms can be visualized in the region up to the axillary level within several minutes. A linear pattern is not imaged in the edematous regions in patients with lymphedema. Instead, no pattern or only a dim stardust or dermal backflow pattern is visualized; these images are thought to reflect retention of ICG in the subcutaneous fat layer^18,19^. In this study, the presence of a splash, stardust or diffuse pattern was taken to indicate lymphedema^20–25^.

### Magnetic Resonance Imaging (MRI)

MRI was performed using an Signa HDxt 1.5 T (GE Healthcare Japan, Tokyo, Japan) and a body coil. The pulse conditions were as follows: short SE (TR400–600 mS, TE29–35 mS); long SE (TR2000 mS, TE80–92 mS); scan frequency, 256; slice thickness, 10–15 mm; and averaging, 1–4 times. The acquisition range was from the lower abdominal region to the bilateral thighs. Partial or complete fluid retention in fat tissue on T2-weighted or STIR imaging was considered to indicate lymphedema.

### Computed Tomography (CT)

Images were obtained with an Aquilion™ 64 scanner (Toshiba Medical Systems, Japan) equipped with a Virtual Place Advance PLUS workstation (AZE, Tokyo, Japan). The acquisition conditions were voltage, 120 kV; current, RealEC; and slice width, 0.5 mm. Lymphedema was diagnosed when a fibrous component was observed in the subcutaneous fat layer.

### Histochemical Evaluation

Samples were obtained from collecting lymphatic channels that did not require anastomosis and were trimmed during LVA surgery. Samples were collected from linear pattern, splash pattern, and mixed stardust and diffuse pattern regions in affected limbs. Skin samples were fixed in 10% neutral phosphate-buffered formalin solution. Subsequently, 4-µm paraffin sections were prepared by the conventional method, subjected to hematoxylin-eosin and Elastica van Gieson staining, and examined under a microscope. In addition, immunostaining was performed using LYVE-1 antibody (Oriental Yeast Corp., Tokyo, Japan). Collecting lymphatic vessels were also examined using transmission electron microscopy (TEM).

## Results

A summary of the 21 cases examined in the study is shown in Table 2. Pitting edema was observed in all patients. The circumference was increased by more than 3 cm in only one patient. On ICG lymphography and MRI, all 21 cases were positive. On lymphoscintigraphy and CT, positive features were noted in 13 and 7 cases, respectively. The sensitivity and specificity of each imaging method are shown in Table 3. The specificity was 1 in all methods, but the sensitivity of ICG lymphography and MRI was superior to that of CT and lymphoscintigraphy. Typical findings from immunostaining of the skin and electron microscopy of the collecting lymph vessels are described below for Case 3, and were found in all cases.

The patient in Case 3 was a 55-year-old woman who underwent mastectomy, axillary lymph node dissection, and chemotherapy and radiotherapy (45 Gy) for right breast cancer 12 years ago. Lymphedema had appeared in the right upper limb 2 years ago and slowly aggravated. Conservative treatment with lymph massage and elastic stockings was used from immediately after the appearance of lymphedema, but edema was not remitted. A physical examination revealed enlargement of the circumference of the right upper extremity by +1.5 cm at the upper arm, +1.0 cm at the elbow, +1.0 cm at the forearm, and +0.5 cm at the wrist, compared to the left upper extremity (Figure 1a,b). Pitting edema was noted in the forearm and medial upper arm. On preoperative lymphoscintigraphy, dermal backflow was noted in the right upper arm over the forearm, compared to normal lymph flow in the healthy left arm, showing mild lymphatic circulatory disorder (Figure 1c). In ICG lymphography (Figure 2, Video S1), several vivid dynamic images of a superficial lymphatic flow pattern in the wrist area were obtained within a few minutes after injection. A splash pattern indicating mild-damaged lymph flow was present in the right forearm and upper arm. A diffuse pattern indicating severely damaged lymph flow was present in the right medial upper arm. As a normal control, ICG lymphography image of contralateral side is shown in Video S2. On MRI, fluid retention was noted in the fat layer of the forearm and medial upper arm. On CT, partial mild fibrosis was apparent in the fat layer of the medial upper arm.

In TEM findings in the linear pattern region, lymph vascular endothelial cells showed a concave structure toward the lumen (Figure 3a) and smooth muscle cells were present in the tunica media as ‘contractile’ smooth muscle cells in collecting lymph vessels (Figure 3d). In the splash pattern area, lymph vascular endothelial cells were flattened (Figure 3b) and smooth muscle cells were altered to a ‘secretory type’ and were present in the tunica media in collecting lymph vessels (Figure 3e). Some ‘secretory’ smooth muscle cells were also diffusely present directly below lymph vascular endothelial cells. In the diffuse pattern region, lymph vascular endothelial cells were flattened in collecting lymph vessels (Figure 3c) and endothelial cells were detached in several regions (Figure 3c, red arrows), exposing collagen fibers of the tunica media to the lumen. Smooth muscle cells of collecting lymph vessels became ‘secretory’, as in the splash pattern area, and were present directly below lymph vascular endothelial cells over the tunica media. The space between smooth muscle cells was widened with overgrowth of collagen fibers (Figure 3f).

In immunostaining (LYVE-1) of the skin in the linear pattern region, capillary lymph vessels were diffusely present directly below the dermis, as in the normal skin (Figure 4a,b,c,e). In the splash and diffuse pattern areas, overgrowth of capillary lymph vessels (Figure 4, red arrows) was noted directly below the dermis and in the middle layer of fat, compared to the normal skin (Figure 4b,d,f).

For further illustration, we describe Case 17. The patient was a 57-year-old woman who underwent mastectomy, axillary lymph node dissection, and chemotherapy and radiotherapy (50 Gy) for left breast cancer 1 year ago. Lymphedema appeared in the right upper limb 10 months ago and slowly aggravated. Conservative treatment with lymph massage and elastic stockings was used from immediately after the appearance of lymphedema, but edema was not remitted. Physical examination revealed enlargement of the circumference of the right upper extremity by +0.5 cm at the upper arm, +0.5 cm at the elbow, +1.0 cm at the forearm, and +0 cm at the wrist, compared to the right upper extremity (Figure 5a,b). On lymphoscintigraphy, dermal back flow (reflux of lymph flow in the skin) was noted in the left upper arm over the forearm, compared to normal lymph flow in the healthy right arm. Lymphoscintigraphy showed normal lymph flow in the bilateral upper arms (Figure 5c). However, an ICG fluorescent test (Figure 6) showed a linear pattern only in one shoulder. A splash pattern was noted in the lateral forearm, lateral upper arm, and right axillary region over the precordia. A diffuse pattern was found in the dorsum of the hand, medial forearm, and medial upper arm. On MRI, mild fluid retention was noted in the lateral forearm. On CT, there was no major change other than an increase in the circumference compared to that of the healthy limb.

## Discussion

In this study, we found that ICG lymphography and MRI were most effective for diagnosis of upper limb lymphedema in a comparison of four diagnostic imaging methods. Regarding the characteristics of these methods, the lymphatic system is observed directly in ICG lymphography and in lymphoscintigraphy, whereas edema in fat tissue is detected in MRI and fibrotic tissue is observed in CT. The most important finding for diagnosis of lymphedema is dermal backflow of lymph from collecting lymph vessels to the skin surface and tissue. A definite diagnosis may be made by ICG lymphography and lymphoscintigraphy because dermal backflow is observed as an abnormality of the lymph vascular system. In contrast, secondary changes in the skin and subcutaneous tissue are observed by MRI and CT, based on which the severity of edema of the affected limb can be determined.

There are also marked differences in direct observation of the lymphatic system between ICG lymphography and lymphoscintigraphy. In ICG lymphography, retained fluid is detectable without background noise because there is no endogenous fluorescence in the near infrared band (780–1500 nm) used for detection of ICG, and this increases the accuracy. The safety of ICG has been shown in clinical practice, and the test does not cause tissue damage [18], [19]. Using lymphoscintigraphy, it is difficult to evaluate lymph vessels in the lateral region of the body because images are acquired only in the anteroposterior direction, whereas observation from multiple directions is possible by moving the camera position in ICG lymphography. The major advantage of ICG lymphography compared to lymphoscintigraphy is the potential for real-time observation of lymph vessels during surgery. However, ICG lymphography cannot be used to observe lymph vessels at more than 2-cm depth in the subcutaneous tissue. In lymphoscintigraphy, labeled albumin is injected and its transport into lymph vessels is observed. Minute subcutaneous lymph vascular abnormalities cannot be detected, but the whole leg can be observed, and this is the only method for observing deep lymph vessels [26]. However, lymphoscintigraphy has disadvantages of radiation exposure, high invasiveness, high costs, and low resolution. Thus, it should be used only in cases that cannot be evaluated by ICG lymphography.

There are also differences in the characteristics of MRI and CT for observing subcutaneous tissue. Both methods can be used to determine the severity of the causative disease in cases of secondary edema and the presence or absence of disease in cases of primary edema, in addition to the presence of lymphedema, by observing the abdominal and intrapelvic regions. Subcutaneous excess water retention can also be observed using MRI. Dilated lymph vessels and the cause of lymph vascular obstruction may also be identified in some cases. However, real-time diagnosis cannot be achieved and the cost is high [26]. On the other hand, overgrowth of fibrous tissue with the progression of lymphedema can be evaluated using CT. Both MRI and CT can be used to evaluate the presence and severity of edema, but differentiation of lymphedema from edema associated with other internal medical diseases is not possible. Thus, to diagnose lymphedema, a lymphatic system-specific ICG test or lymphoscintigraphy is required.

We also performed biopsy of the skin and collecting lymph vessels in the limb with lymphedema at the same time as the diagnostic imaging. No histological changes were noted in regions with a linear pattern in ICG lymphography, but flattening of collecting lymph vascular endothelial cells and overgrowth of capillary lymph vessels were found in areas with a splash pattern. In the advanced stage, which shows a diffuse pattern, lymph vascular endothelial cells were detached and collagen fibers had accumulated. In Case 17, the findings were normal on lymphoscintigraphy, but a splash pattern, stardust pattern, and diffuse pattern were observed in ICG lymphography and histological changes had occurred in these regions. This suggests that when an abnormal image is obtained in ICG lymphography, but the image is normal in lymphoscintigraphy, the finding is not likely to be a false positive in the ICG test, but more likely to be a false negative in lymphoscintigraphy.

Different diagnostic imaging methods for lymphedema have both advantages and disadvantages, and therefore several tests should be performed for one patient. The following diagnostic flow may be useful: i) examination of the lymphatic system using ICG, ii) evaluation of edema in fat tissue by MRI, and iii) design of treatment and a surgical plan based on this information. A further study is required to determine the effectiveness of this diagnostic and therapeutic strategy.

In conclusions, the combination of ICG lymphography and MRI may provide a more effective imaging method for definite diagnosis of lymphedema, which cannot be achieved with lymphoscintigraphy alone.

## Supporting Information

Video S1 **ICG lymphography of affected side (case3).**
(WMV)

Video S2 **ICG lymphography of unaffected side (case 3).**
(WMV)
